# Supplementary material for: Content-rich biological network constructed by mining PubMed abstracts
Source: BMC Bioinformatics. 2004 Oct 8;5:147. doi: 10.1186/1471-2105-5-147 (PMC528731; doi:10.1186/1471-2105-5-147)
Supplement: Additional File 2 — The original results of the above study (non-essential files are deleted to keep the file size under the limit set by BMC bioinformatics). [file 1471-2105-5-147-S2.bz2 › chilibotAdditionalFile2/dip40/337ID8621492E4139/html/LTA_LTB.html]

 


 **LTA** and **LTB** 
  
Found 137 abstracts in PubMed, retrieved 40.  
 

 What does Google say? 
 PDF only 
| .edu only 

---

**Interactive relationship** (e.g. stimulation, inhibition, etc)

**Inhibitory relationship**- In vitro experiments suggested that the intracellullar accumulation of LTC 4 in mrp1 mice results in product inhibition of LTC 4 synthase, diminishing substrate competition between  **LTA**  4 hydrolase which yields  **LTB**  4 and LTC 4 synthase for the available  **LTA**  4.  Ref: 11238654 J Immunol, 2001
- SC 57461A is a competitive, selective, and orally active inhibitor of  **LTA**  4 hydrolase in vivo, making it useful to explore the contribution of  **LTB**  4 to a number of inflammatory diseases.  Ref: 11805220 J Pharmacol Exp Ther, 2002
- Additional structure activity relationship studies around this structural class resulted in the identification of a series of alpha, beta, and gamma amino acid analogues that are potent inhibitors of the  **LTA**  4 hydrolase enzyme and demonstrated good oral activity in a mouse ex vivo whole blood  **LTB**  4 production assay.  Ref: 12139459 J Med Chem, 2002
- SC 57461A 3 methyl 3 4 phenylmethyl phenoxy propyl amino propanoic acid HCl is a potent inhibitor of human recombinant  **LTA**  4 hydrolase epoxide hydrolase and aminopeptidase activities, K i values = 23 and 27 nM, respectively as well as calcium ionophore induced  **LTB**  4 production in human whole blood IC 50 = 49 nM.  Ref: 11805220 J Pharmacol Exp Ther, 2002
**Neutral relationship**- The endogenous  **LTA**  4 hydrolase form the  **LTB**  4 from  **LTA**  4 in erythrocytes, platelet, endothelial or smooth muscle cells.  Ref: 12208150 Med Hypotheses, 2002
- **LTA**  4 is further metabolized to either LTC 4 or  **LTB**  4 by the enzyme LTC 4 synthase or  **LTA**  4 hydrolase, respectively.  Ref: 12021253 J Clin Invest, 2002
- As the rate limiting step for  **LTB**  4 production,  **LTA**  4 hydrolase represents an attractive target for therapeutic agents that interfere with  **LTB**  4 production.  Ref: 11805219 J Pharmacol Exp Ther, 2002
- LT consists of soluble LT alpha  [ **LTA** ]  3, which binds to TNF R1 and TNF R2, and membrane LT alpha  [ **LTA** ]  1 beta 2, which binds to LT beta  [ **LTB** ]  R.  Ref: 12882833 Int Immunol, 2003
- Transplantation of lymphoid tissues from LT beta  [ **LTB** ]  mice, which have cervical and mesenteric lymph nodes, into LT alpha  [ **LTA** ]  mice, which do not, did not alter the incidence of CNS scrapie.  Ref: 11932402 J Virol, 2002
- Furthermore, LT alpha  [ **LTA** ]  mice had a higher incidence and shorter incubation period for developing disease following inoculation than did LT beta  [ **LTB** ]  mice.  Ref: 11932402 J Virol, 2002
- This observation previously has led to the suggestion that  **LTA**  4 produced by leukocytes may, in some cases, be delivered to other cell types before being converted into LTC 4 or  **LTB**  4.  Ref: 12021253 J Clin Invest, 2002
- The lymphotoxin beta  [ **LTB** ]  receptor LT beta  [ **LTB** ]  R, a member of the tumor necrosis factor TNF receptor family, plays a crucial role in lymphoid organogenesis by signaling through its functional ligand LT alpha  [ **LTA** ]  1 beta 2.  Ref: 11399522 Eur Cytokine Netw< MedlineTA>Eur Cytokine Netw, 2002

**Non-interactive relationship** (e.g. studied together, co-existance, homology, etc.)

- The enzyme  **LTA**  4 hydrolase represents an attractive target for pharmacological intervention in these disease states, since the action of this enzyme is the rate limiting step in the production of  **LTB**  4.  Ref: 12139459 J Med Chem, 2002
- Lymphotoxin alpha  [ **LTA** ] , lymphotoxin beta  [ **LTB** ] , C C chemokine ligand CCL 21, CXC chemokine ligand CXCL 12, CXCL13, and CCL22 were increased in thyroids from autoimmune patients.  Ref: 12794165 J Immunol< MedlineTA>J Immunol, 2002
- Lymphotoxin alpha  [ **LTA** ]  and lymphotoxin beta  [ **LTB** ]  deficient mice differ in susceptibility to scrapie evidence against dendritic cell involvement in neuroinvasion.  Ref: 11932402 J Virol, 2002
- Further, it is possible that an as yet unidentified cell found more abundantly in LT alpha  [ **LTA** ]  than in LT beta  [ **LTB** ]  mice may assist in the amplification of scrapie infection in the periphery and favor susceptibility to CNS disease following peripheral routes of infection.  Ref: 11932402 J Virol, 2002
- There was relatively little demyelination associated with inflammation in LT alpha  [ **LTA** ]  and LT beta  [ **LTB** ]  R mice compared to susceptible SJL mice, suggesting the possibility that LT plays a role in mediating demyelination.  Ref: 12882833 Int Immunol, 2003
- The proportions of the produced leukotriene A 4  **LTA**  4 hydrolates sum of 6 trans  **LTB**  4 and 12 epi 6 trans  **LTB**  4,  **LTB**  4 and cysteinyl leukotriene were approx.  Ref: 11772398 Biochem J, 2002
- Homozygotes for the  **LTA**  NcoI TNFc  [ **LTB** ]  aa13L aa26 haplotype 1 1 1 1 did not respond P overall = 0.007.  Ref: 11313304 Gastroenterology, 2001
- ... the degraded product of  **LTA**  trans  **LTB**  4, was doubled in BMMC from LTC 4 S mice because of lack of utilization.  Ref: 11319240 J Biol Chem, 2001
- This activity of LT depends on membrane LT alpha  [ **LTA** ]  1 beta 2 and not soluble LT alpha  [ **LTA** ]  3, since DA virus persisted and induced inflammatory demyelination in LT beta  [ **LTB** ]  R, but not TNF R1 or TNF R2, mice.  Ref: 12882833 Int Immunol, 2003
- Functional studies in gene knockout and transgenic mice systems have shown that lymphotoxin alpha  [ **LTA** ]  and lymphotoxin beta  [ **LTB** ]  LT alpha  [ **LTA** ]  and LT beta  [ **LTB** ]  are of fundamental importance in peripheral lymphoid organ development, but it remains unclear what role these cytokines have to play in the adult immune response and in the pathogenesis of disease.  Ref: 12474234 J Pathol, 2003
- Expression of IL 1alpha, IL 1beta, IL 6, IL 10, IL 12, IFN beta, IFN gamma, LT beta  [ **LTB** ] , TGF beta, TNF a and TNF beta  [ **LTA** ]  mRNA levels in CRT MG human astroglioma cells upon Fas ligation was investigated using RNase protection assay RPA.  Ref: 11949822 J Neurooncol, 2002
- Leukotriene LT A 4 hydrolase is a dual function enzyme that is essential for the conversion of  **LTA**  4 to  **LTB**  4 and also possesses an aminopeptidase activity.  Ref: 11805220 J Pharmacol Exp Ther, 2002
- Particular alleles of the tumor necrosis factor gene cluster TNFA,  **LTA** ,  **LTB**  are known to be associated with peculiar clinical forms of sarcoidosis.  Ref: 12102613 Sarcoidosis Vasc Diffuse Lung Dis, 2002
- Although the essential role of tumor necrosis factor TNF in the control of intracellular bacterial infection is well established, it is uncertain whether the related cytokines lymphotoxin alpha  [ **LTA** ]  LTalpha3 and lymphotoxin beta  [ **LTB** ]  LTbeta have independent roles in this process.  Ref: 11208864 J Exp Med, 2001
- In addition, the observation of striking similarities between the mRNA accumulation kinetics of LT alpha  [ **LTA** ]  and LT beta  [ **LTB** ]  during these treatments indicates tight coupling of expression under certain conditions.  Ref: 11747624 J Interferon Cytokine Res, 2001
- Samples homozygous at TNFA 308 and BAT1 intron 10 demonstrated identity between the African TNFA 308\*2 haplotype, the 8.1AH and the Asian diabetogenic 58.1AH HLA A33, B58, DR3 across a region spanning BAT1, ATP6G, IKBL,  **LTA** , TNFA,  **LTB** , LST 1 and AIF 1.  Ref: 12859597 Tissue Antigens, 2003
- We investigated leukotriene A 4  **LTA**  4 hydrolase expression and  **LTB**  4 levels in oral epithelium in relation to the clinical disease manifestation and immunohistopathology and  **LTA**  4 hydrolase expression in cultured oral keratinocytes.  Ref: 12070603 Virchows Arch, 2002
- Our data demonstrate an upregulation of LT beta  [ **LTB** ]  mRNA by the inflammatory cytokines TNF and LT alpha  [ **LTA** ] .  Ref: 11747624 J Interferon Cytokine Res, 2001
- The LT alpha  [ **LTA** ]  and LT beta  [ **LTB** ]  R mice failed to mount a virus specific cytotoxic T cell response.  Ref: 12882833 Int Immunol, 2003
- We used mice lacking Peyer s patches and or lymph nodes because of lymphotoxin alpha  [ **LTA** ]  gene deficiency or treatment in utero with lymphotoxin beta  [ **LTB** ]  receptor IgG and tumor necrosis factor receptor I 55 IgG fusion proteins.  Ref: 12466141 Am J Pathol, 2002
- LT alpha  [ **LTA** ]  deficient mice and LT beta  [ **LTB** ]  R Fc transgenic mice were profoundly susceptible to murine CMV infection.  Ref: 11672543 Immunity, 2001
- ... the  **LTA**  4 hydrolase inhibitor SC57461A and the LT biosynthesis inhibitor MK 0591 inhibited AA but not  **LTB**  4 mediated PLD activation.  Ref: 12660228 J Leukoc Biol, 2003
- FDC generation and maintenance in germinal centers is dependent on lymphotoxin alpha  [ **LTA** ]  LT alpha  [ **LTA** ]  and LT beta  [ **LTB** ]  signaling components.  Ref: 11932402 J Virol, 2002
- There are five types of LT  **LTA**  4,  **LTB**  4, LTC 4, LTD 4 and LTE 4.  Ref: 11772331 Expert Opin Pharmacother, 2002
- Neither lymphotoxin alpha  [ **LTA** ]  nor lymphotoxin beta  [ **LTB** ]  receptor expression is required for biogenesis of lymphoid aggregates or differentiation of natural killer cells in the pregnant mouse uterus.  Ref: 12603600 Immunology, 2003
- Human placentae and two of the cell types in placentae cytotrophoblasts and macrophages were examined by RT PCR for transcripts of the eight TNF superfamily ligands known to induce death of activated immune cells, tumour cells, and virus infected cells TNFalpha, LT alpha  [ **LTA** ] , LT beta  [ **LTB** ] , FasL, TRAIL, TWEAK, LIGHT, 4 1BBL.  Ref: 11597186 Placenta< MedlineTA>Placenta, 2003
- Cyclosporin A blocks the expression of lymphotoxin alpha  [ **LTA** ] , but not lymphotoxin beta  [ **LTB** ] , in human peripheral blood mononuclear cells.  Ref: 12176893 Blood, 2002
- We report here that by the oral route, LT alpha  [ **LTA** ]  mice developed scrapie while LT beta  [ **LTB** ]  mice did not.  Ref: 11932402 J Virol, 2002
- Lymphotoxin beta  [ **LTB** ]  LT beta  [ **LTB** ]  is a tumor necrosis factor TNF related membrane bound cytokine that forms a heterotrimeric surface lymphotoxin LT complex with LT alpha  [ **LTA** ]  on the surface of lymphoid cells.  Ref: 11747624 J Interferon Cytokine Res, 2001
- Genomic DNA was extracted, followed by polymerase chain reaction amplification and sequencing for two promoter TNF alpha variants at positions 238 and 308, as well as restriction fragment length analysis for four polymorphic loci within the TNF beta  [ **LTA** ]  gene NcoI, TNFc  [ **LTB** ] , aa13, aa26.  Ref: 11922568 Am J Gastroenterol, 2002
- Expression of cytokines TNF beta  [ **LTA** ]  and LT beta  [ **LTB** ]  preceded, and the expression of TGF beta1 followed chemokine upregulation.  Ref: 12828562 Scand J Immunol, 2003
- LT alpha  [ **LTA** ] , LT beta  [ **LTB** ] , LT betaR, and LT alpha  [ **LTA** ]  x TNFalpha mice resisted infection and contained no infectivity in spleens and lymph nodes when present.  Ref: 11792852 Proc Natl Acad Sci U S A, 2002
- METHODS, Three TNF microsatellite loci TNFa, TNFb  [ **LTA** ] , TNFc  [ **LTB** ] , the TNF 308 polymorphism, the IL 10.G microsatellite locus, and 3 i allelic polymorphisms in the IL 10 5 region were typed using PCR in 135 acute pancreatitis patients and ethnically matched normal controls n=107.  Ref: 11852287 JOP, 2000
- Although term cytotrophoblasts contained mRNAs specific for TNF alpha, LT alpha  [ **LTA** ] , TWEAK, and 4 1BBL, messages encoding LT beta  [ **LTB** ] , FasL, TRAIL, and LIGHT were absent.  Ref: 11597186 Placenta< MedlineTA>Placenta, 2000
- This study suggests that  **LTA**  4 hydrolase expression and elevated  **LTB**  4 levels in oral mucosal epithelium are integral parts of the induction and progression of chronic inflammatory reactions.  Ref: 12070603 Virchows Arch, 2002
- To investigate LT alpha  [ **LTA** ]  beta s activities in lymphoid organogenesis, mice simultaneously expressing LT alpha  [ **LTA** ]  and LT beta  [ **LTB** ]  under rat insulin promoter II RIP control were compared with RIPLT alpha mice in a model of lymphoid neogenesis and with LT beta  [ **LTB** ]  mice.  Ref: 12732657 J Exp Med, 2003
- induced the expression of granulocyte macrophage colony stimulating factor GM CSF and G CSF by 5 h and the expression of additional cytokines, including TGF beta, TNF beta  [ **LTA** ] , lymphotoxin beta  [ **LTB** ]  LT beta  [ **LTB** ] , interferon gamma IFN gamma, and IFN beta by 40 h post burn infection.  Ref: 11792126 Cytokine, 2001
